# An engineered channelrhodopsin optimized for axon terminal activation and circuit mapping

**DOI:** 10.1038/s42003-021-01977-7

**Published:** 2021-04-12

**Authors:** Shun Hamada, Masashi Nagase, Tomohiko Yoshizawa, Akari Hagiwara, Yoshikazu Isomura, Ayako M. Watabe, Toshihisa Ohtsuka

**Affiliations:** 1 Department of Biochemistry, Faculty of Medicine, University of Yamanashi, Yamanashi, Japan; 2 Institute of Clinical Medicine and Research, Research Center for Medical Sciences, The Jikei University School of Medicine, Chiba, Japan; 3 Department of Physiology and Cell Biology, Graduate School of Medical and Dental Sciences, Tokyo Medical and Dental University, Tokyo, Japan; 4 Brain Science Institute, Tamagawa University, Tokyo, Japan

**Keywords:** Synaptic transmission, Molecular neuroscience

## Abstract

Optogenetic tools such as channelrhodopsin-2 (ChR2) enable the manipulation and mapping of neural circuits. However, ChR2 variants selectively transported down a neuron’s long-range axonal projections for precise presynaptic activation remain lacking. As a result, ChR2 activation is often contaminated by the spurious activation of *en passant* fibers that compromise the accurate interpretation of functional effects. Here, we explored the engineering of a ChR2 variant specifically localized to presynaptic axon terminals. The metabotropic glutamate receptor 2 (mGluR2) C-terminal domain fused with a proteolytic motif and axon-targeting signal (mGluR2-PA tag) localized ChR2-YFP at axon terminals without disturbing normal transmission. mGluR2-PA-tagged ChR2 evoked transmitter release in distal projection areas enabling lower levels of photostimulation. Circuit connectivity mapping in vivo with the Spike Collision Test revealed that mGluR2-PA-tagged ChR2 is useful for identifying axonal projection with significant reduction in the polysynaptic excess noise. These results suggest that the mGluR2-PA tag helps actuate trafficking to the axon terminal, thereby providing abundant possibilities for optogenetic experiments.

## Abstract (web summary)

Hamada et al. engineer and utilise a channelrhodopsin-2 variant that is localized to presynaptic axon terminals. They demonstrate its use for circuitry mapping in vivo and thus provide a useful tool for future optogenetic experiments

## Introduction

Over the past decade, genetically targeted manipulation of neuronal activity with light, termed optogenetics^1^, has revolutionized neuroscience strategies^2–4^. The light-activated cation channel, channelrhodopsin-2 (ChR2), from *Chlamydomonas reinhardtii* and its variants are now widely used optogenetic actuators that depolarize the membrane potential enough to elicit action potentials (spikes) upon blue light illumination^5–7^. The cell-type- or pathway-specific expression of ChR actuators offers a flexible manipulation of neural activity, to elucidate various functional neural circuits, including the olfactory system^8,9^. ChRs’ selective effects on neurons or circuits can change animal behaviors^10^ and will potentially alleviate neuropsychiatric diseases^11^.

Optogenetic manipulation may evolve further by addition of localization tags to subcellular compartments^7^. Insertion of the Golgi trafficking signal after ChRs increased total photocurrents because of dynamic expression on the plasma membrane^12^. Somatodendritic expression was enhanced by the myosin Va-binding domain^13^, or enriched with the targeting motif of Kv2.1^14^ or neuroligin-1^15^. More specifically, the PDZ domain-binding motif, ETQV, concentrated ChR2 in the postsynaptic density^16^. While these modifications are beneficial for activating target neurons more locally and efficiently, activation of long-range axonal projections is not well developed. Localization of ChR2 to the axon initial segment (AIS) by adding the ankyrin G-binding domain of the sodium channel, Nav1.2^17^ or Nav1.6^18^, unexpectedly disrupted endogenous Nav clusters at the AIS, which suppressed spike generation after ChR2 photoactivation^17–19^. Fusion of ChR2 with a myosin VI binding domain (MVIBD) promoted light-evoked spike generation in local axons near the soma^20^, but evaluation of long-range axons was not extensively performed^7^. In songbird studies, ChR2 with a neurexin 1α tag promoted presynaptic enrichment, however, these reports did not describe validation of functional improvement of the channels.^21,22^. Thus, it is still worth developing novel ChR2 tools specific to long-range axons and axon terminals that will be capable of eliciting spikes and synaptic release.

Optogenetically, axonal projections can be identified by detecting “antidromic spikes” from the projection area^23–25^. However, in transgenic animals expressing ChR2 widely across areas, the photostimulation activates somata, dendrites, or passing axons of untargeted neurons according to their anatomical arrangements, which causes unexpected spikes. Therefore, not only subcellular enrichment of ChR2 to distal axons and axon terminals, but also lowering expression in somata and dendrites would be strategically beneficial for triggering antidromic spikes and synaptic release specifically in intricate neural circuits. However, such axon-oriented ChR2 variants have not been established so far.

Here, we showed that ChR2 was effectively transported to axon terminals of cortical and subcortical projection neurons by fusing it with the C-terminal domain of metabotropic glutamate receptor 2 (mGluR2), which localizes at perisynaptic areas^26^. Furthermore, its excess expression and light-evoked spike generation at the soma and dendrites were suppressed by adding a proteolytic motif [PEST, ref. ^27^] and an axon-targeting element [ATE, ref. ^28^] to the mGluR2 domain (mGluR2-PA tag). Importantly, the light-evoked presynaptic activation drove normal synaptic transmission, suggesting the addition of the localization signals to the ChR2 did not disrupt the basal synaptic transmission properties. Finally, the newly developed ChR2-yellow fluorescence protein (YFP)-mGluR2-PA successfully evoked antidromic spikes in the spike collision test^23,24^. Taken together, the presynapse localization tag, mGluR2-PA, will make the optogenetic tool more powerful for preferentially stimulating axonal projections as well as for controlling synaptic transmission in vivo and in vitro.

## Results

### Axonal localization of ChR2-YFP using presynaptic-interacting domains

**Fig. 1:**
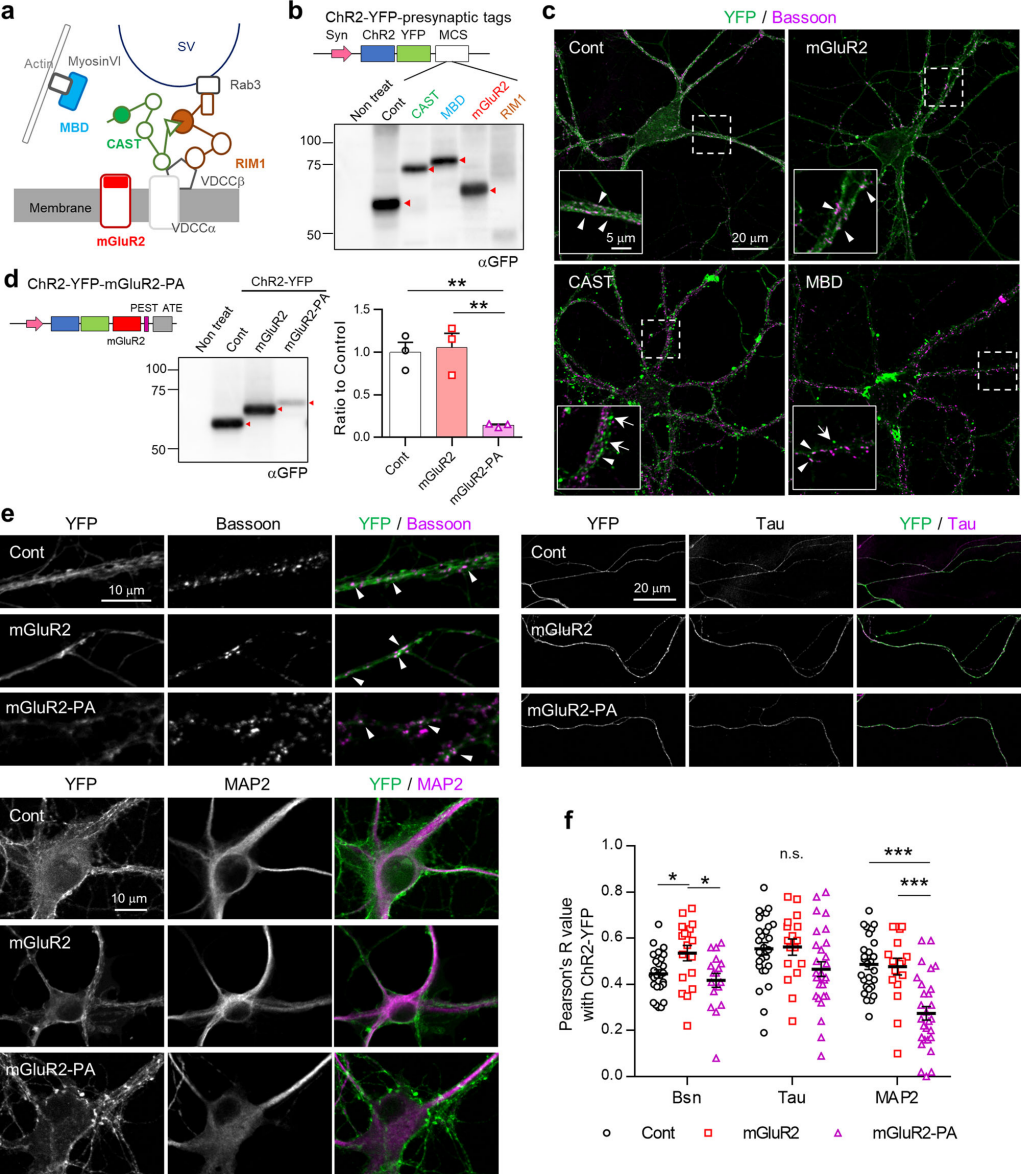
Screening of presynaptic-localizing tags. **a**, **b** Scheme of presynaptic targeting strategy. Presynaptic-localizing tags were inserted in the multi cloning site (MCS) following the ChR2-YFP sequence. Syn; human *synapsin 1* promoter. Western blot of ChR2-YFP with presynapse tags in primary hippocampal neurons. ChR2-YFP-RIM1 showed no signal. **c** The expression and localization of ChR2-YFP with presynapse tags were identified in primary hippocampal neurons by immunocytochemistry. Arrowheads indicate the presynaptic terminal colocalized with Bassoon and arrows indicate the dotty aggregates. Dashed line regions were expanded in inlets. **d** Insertion of the PEST and ATE sequences. The expression of ChR2-YFP-mGluR2-PA was significantly lower than that of the control and mGluR2 tag. Results are mean ± SEM; *n* = 3 independently prepared samples. **P < 0.01 (one-way ANOVA followed by post hoc Tukey’s test). **e** The distribution of control ChR2-YFP, -mGluR2, and -mGluR2-PA was determined by immunocytochemistry with the indicated antibodies, Bassoon, Tau, and MAP2. **f** Compared with the control, the colocalization of ChR2-YFP with Bassoon as indicated by the R-value was significantly increased with the mGluR2 tag, whereas with Tau it was not significantly different. Compared with the other two groups, the colocalization with MAP2 was significantly reduced with the mGluR2-PA tag. Bassoon staining: Control, *n* = 26 cells; mGluR2, *n* = 18; mGluR2-PA, *n* = 17. Tau and MAP2 staining: Control, *n* = 28; mGluR2, *n* = 17; mGluR2-PA, *n* = 29. Results are mean ± SEM; *P < 0.05, ***P < 0.001 (one-way ANOVA with post hoc Bonferroni’s multiple comparison test).

For subcellular localization of the optogenetic actuator, ChR2, to the presynaptic terminal, interacting domains of presynaptic proteins were fused to ChR2(H134R)-EYFP (hereafter, “control ChR2-YFP”) using a modified multi cloning site (MCS) (Fig. 1a, b). We chose two presynaptic proteins, CAST and RIM1, which organize the synaptic vesicle release at the active zone (AZ) (Fig. 1a)^29,30^. As their full length was too large to insert as localization tags, we attempted to use the putative N-terminal coiled-coil domain of CAST (139–306 aa; ref. ^31^) and the RIM1 PDZ domain (614–693 aa), which is essential for interacting with CAST^31,32^ at the presynaptic terminal. We also applied the C-terminal region of mGluR2 (820–872 aa), as mGluR2 has been demonstrated to localize at the preterminal, i.e., the perisynaptic terminal portion of axons^26^. These tags were evaluated together with the MVIBD tag, derived from a fusion of optineurin (420–524 aa) and DAB2 (429–516 aa) as reported previously^20^. First, the expression of ChR2-YFP with presynaptic localization tags was evaluated in primary cultured hippocampal neurons using an adeno-associated virus (AAV) vector (serotype DJ; AAV-DJ). Judging from the band size, the expression of ChR2-YFP was preserved with the CAST, mGluR2, and MVIBD tags, whereas, unexpectedly, it was attenuated with the RIM1 tag (Fig. 1b, Supplementary Fig. 1a). We further investigated the localization of ChR2-YFP with these presynaptic tags in cultured neurons. While control ChR2-YFP was widely diffused on the membranes of the soma, dendrites, and axons, the signal of ChR2-YFP with presynaptic tags looked relatively punctate (Fig. 1c). Unfortunately, CAST and MVIBD tags displayed dotty signals in somata and axon shafts, which were bigger than those of the AZ marker, Bassoon, probably because of undesirable aggregation. Thus, we decided to focus on further evaluation and improvement of the mGluR2 tag.

### Suppressed somatodendritic expression of mGluR2-PA-tagged ChR2-YFP

Although the mGluR2-tagged ChR2-YFP showed punctate signals that were colocalized with Bassoon, the fluorescent signal was still abundant on the soma-and dendrites-like membrane structures (Fig. 1c). Insertion of PEST^27^ and ATE^28^ after the ChR2-YFP-mGluR2 significantly suppressed its expression down to 20% compared with that of the control or mGluR2 tag alone (Fig. 1d, Supplementary Fig. 1b). Note that the C-terminal PDZ-binding motif^33^ was ablated by the insertion of the PEST and ATE sequences into the MCS.

To quantify the specific localization of mGluR2-tagged ChR2-YFP, the spatial distribution of YFP signals was compared with that of immunoreactivities to anti-Bassoon, anti-Tau (axon marker), and anti-MAP2 (dendritic marker), using the colocalization index, Pearson’s R-value (Fig. 1e, f; see Online Methods). The presynaptic colocalization of ChR2-YFP and Bassoon was enhanced by the mGluR2 tag compared with the control, while the mGluR2-PA tag maintained the colocalization at a similar level to the control despite the proteolytic effect of the PEST sequence (Fig. 1f). The mGluR2-PA tag also preserved the axonal colocalization with Tau, possibly because of axonal trafficking by the ATE sequence. However, the mGluR2-PA tag largely decreased the dendritic colocalization with MAP2 because of the PEST proteolysis. Therefore, we think the mGluR2-PA tag is a promising candidate for localizing ChR2-YFP at distal axons and axon terminals.

### In vivo presynaptic localization of mGluR2-PA-tagged ChR2-YFP

**Fig. 2:**
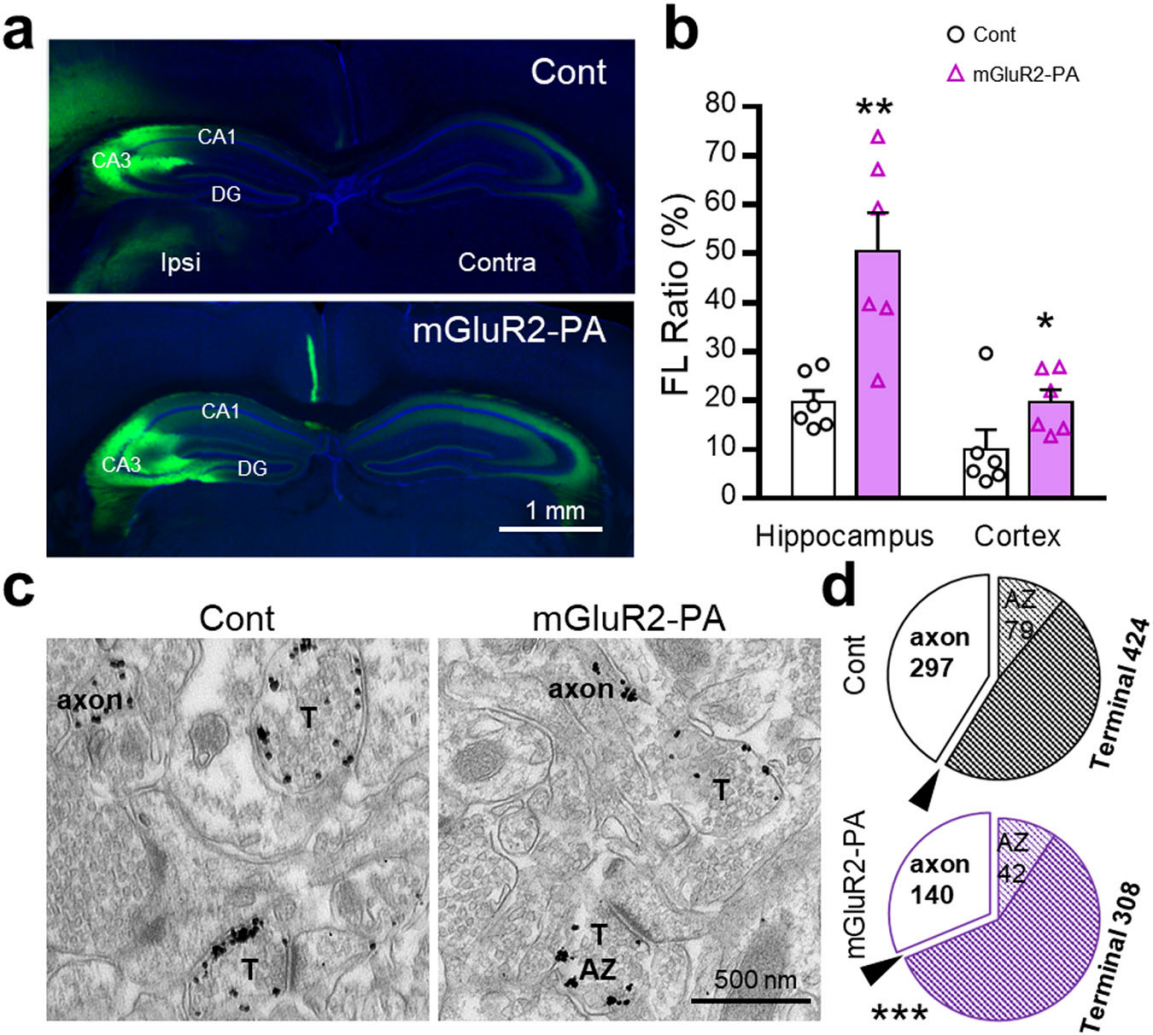
In vivo expression and ChR2-YFP localization to the contralateral projection terminal. **a** Injection of AAV to express ChR2-YFP (Cont) or mGluR2-PA in the hippocampal CA3 neurons (Ipsi), which distributed the projection fibers to the contralateral hippocampus (Contra). **b** The fluorescence intensity at the projection site was normalized by the intensity at the injection sites in the commissural hippocampal and cortical pathways. The normalized contralateral expression was significantly increased with mGluR2-PA. The results are mean ± SEM; *n* = 6 (two sections each from three mice), *P < 0.05, **P < 0.01, student’s *t*-test. **c** Immunoelectron microscopy demonstrated the expression of ChR2-YFP as detected by anti-GFP antibody at the contralateral hippocampus after injection of ChR2-YFP (Cont) and mGluR2-PA. Immunogold signals, black dots, were detected either on the membrane of the presynaptic terminal (T) including more than five synaptic vesicles or on the axon components. If a signal was detected on the synaptic membrane, the terminal was further classified into the active zone (AZ). **d** The profile of immunopositive structures in three mice of each AAV. Statistical analysis using the 2 × 2 χ^2^ test evaluated the accumulation of ChR2-YFP-mGluR2-PA at the terminal (Cont; terminal = 424, axon = 297, mGluR2-PA; terminal = 308, axon = 140, *P* = 0.0006), while distribution at the AZ within the terminal components was not significantly different (Cont: AZ = 79, non-AZ = 345; mGluR2-PA: AZ = 42, non-AZ = 266, *P* = 0.072).

In addition to the in vitro observation in primary cultured neurons, we investigated the subcellular localization of ChR2-YFP in projection neurons of the mouse hippocampus and cortex in vivo (Fig. 2a, b, and Supplementary Fig. 2). The AAV vector containing ChR2-YFP alone or with the mGluR2-PA tag was injected unilaterally into the hippocampal CA3 area, and the expression in the contralateral projection area was compared. The CA3 pyramidal cells in the injection site showed somatic expression of ChR2-YFP, which was reduced by adding the mGluR2-PA tag to ChR2-YFP (Supplementary Fig. 2a). In contrast, their axonal projection in the contralateral CA3 area showed relatively stronger expression of ChR2-YFP in the mGluR2-PA group than in the control (Fig. 2a, Supplementary Fig. 2a). Consequently, the ratio of fluorescence intensity (FL) in the projection area (contralateral) to that in the injection area (ipsilateral) was significantly higher in the presence of the mGluR2-PA tag (Fig. 2b). Similarly, the FL ratio was significantly increased by mGluR2-PA in the motor cortex (Fig. 2b, Supplementary Fig. 2b). Using our AAV vector, we did not find ectopic ChR2-YFP expression in somata and dendrites at the projection area of CA3 and M1 (Fig. 2, Supplementary Fig. 2). Furthermore, we examined whether indeed ChR2-YFP accumulates at the presynaptic terminals rather than the axon shafts within the contralateral projection area using immunoelectron microscopy. Immunogold particles identifying ChR2-YFP with anti-GFP antibody were detected on the membrane of axons and presynaptic terminals on the contralateral side (Fig. 2c). The ChR2-YFP signals were mostly at the presynaptic terminals, and the mGluR2-PA tag effectively accumulated them at the terminal (Fig. 2d; *P* < 0.001 χ^2^ test, terminals vs. axons by control vs. mGluR2-PA). The immunogold signal at the AZ structure of the presynaptic terminal was not significantly different between control and mGluR2-PA (Fig. 2d), consistent with its non-AZ distribution^26^. These results confirmed that the mGluR2-PA tag effectively decreased the ChR2-YFP expression at the soma and dendrites, while it preserved the expression at distal axons and axon terminals.

### Physiological validation of ChR2-YFP with presynaptic tags

**Fig. 3:**
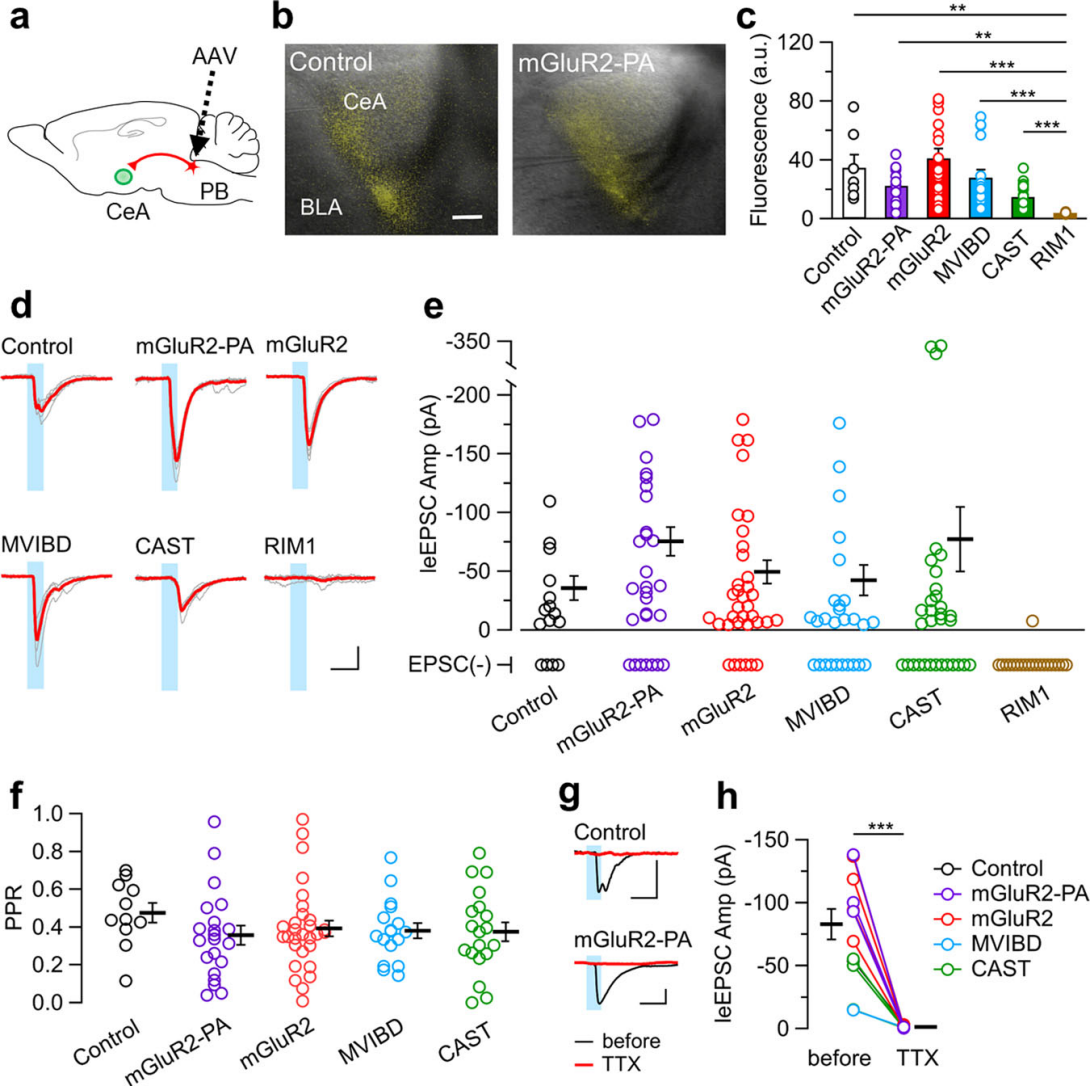
Functional validation of ChR2 with presynaptic-localizing tags. **a** A schematic of the long-projecting pathway from the brain stem to the amygdala (PB, parabrachial nucleus; CeA, central amygdala). **b** Representative images of the bright field with oblique illumination merged with ChR2-YFP fluorescent images of control and mGluR2-PA in the projection sites. BLA, basolateral amygdala. Scale bar, 100 μm. **c** Summary of YFP fluorescence intensities in the CeA, the capsular part (control, *n* = 7 slices; mGluR2-PA, *n* = 15; mGuR2, *n* = 17; MVIBD, *n* = 16; CAST, *n* = 18; RIM1, *n* = 11). The expression of control ChR2 was adjusted by AAV dilution to a level similar to that of ChR2 with tags. ***P < 0.001, **P < 0.01 (Kruskal–Wallis test followed by post hoc Steel–Dwass test). **d** Representative traces of EPSCs (gray, six consecutive responses; red, average) evoked by photostimulation (every 20 s, duration 5 ms, intensity 27 mW/mm^2^; blue) in each group. Scale bars, 50 pA and 10 ms. **e** Summary of the leEPSC amplitudes in neurons evoked by photostimulation (control, *n* = 11 from 15 recorded cells; mGluR2-PA, *n* = 21/28; mGluR2, *n* = 29/35; MVIBD, *n* = 17/27; CAST, *n* = 19/32; RIM1, *n* = 1/20). There was no significant difference in the amplitude between groups (*P* = 0.0793, Kruskal–Wallis test). The bottom plots indicate recorded neurons without leEPSCs. **f** Summary of paired-pulse ratio (PPR) (control, *n* = 11 cells; mGluR2-PA, *n* = 21; mGluR2, *n* = 29; MVIBD, *n* = 17; CAST, *n* = 19). There was no significant difference (*P* = 0.3404, Kruskal–Wallis test). **g** Representative averaged traces of leEPSC (*n* = 15 traces) before (black) and during (red) tetrodotoxin (TTX) application in the control and mGluR2-PA cells. Scale bars, 50 pA and 10 ms. **h** The effect of TTX on the leEPSC amplitude (control, *n* = 1 cell; mGluR2-PA, *n* = 3; mGluR2, *n* = 5; MVIBD, *n* = 1; CAST, *n* = 3). TTX completely abolished the leEPSC. ***P < 0.001 (Welch’s *t*-test). Scale bars, 50 pA and 10 ms.

While the mGluR2-PA tag successfully accumulated ChR2 at presynaptic terminals as described above, the manipulation may obstruct the ChR2 function per se or the endogenous machinery of presynaptic vesicular release. Therefore, it was crucial to validate the neuronal functions when using ChR2 with presynaptic tags including mGluR2-PA. To this end, we expressed these ChR2-tag variants in glutamatergic neurons in the parabrachial nucleus (PB) of the pons using the AAV vectors in vivo, and analyzed the synaptic response in the central amygdala (CeA) neurons by photostimulating the axon terminals of the long-range PB-CeA pathway in acute brain slices (Fig. 3a and Supplementary Fig. 3a–c). As observed in the cultured neurons, ChR2 with presynaptic tags, except for the RIM1 tag, were expressed in the projection site (Fig. 3b, c). In this experimental condition, control ChR2-YFP tended to be overexpressed (Supplementary Fig. 3b, c using the original AAV titer). To better compare the ChR2 functions among the tag groups, we adjusted the titer of the AAV vector with the control ChR2-YFP to a similar expression level at the projection site by diluting the AAV titer (Fig. 3b, c). Light-evoked excitatory postsynaptic currents (leEPSCs) were obtained from the CeA neurons for the ChR2 with presynaptic tags (Fig. 3d, e). The RIM1 tag variant showed poor expression and almost no leEPSC (Fig. 3d, e, and Supplementary Fig. 3d). The proportion of recorded cells that displayed leEPSCs was not significantly different among the control, mGluR2-PA, MVIBD, and CAST groups, but a larger population displayed leEPSCs in the mGluR2 group (Fig. 3e and Supplementary Fig. 3d). Furthermore, the amplitude of leEPSCs was not significantly different among the control, mGluR2-PA, mGluR2, MVIBD, and CAST groups, but large leEPSCs were more frequently observed in the mGluR2-PA group compared with the other groups (Fig. 3e). The leEPSCs average in the CAST group was high because three neurons untypically exhibited very large leEPSCs (>−300 pA), which had a long latency and/or low reliability to high frequent photostimulation (Supplementary Fig. 3e). While the kinetics, including the latency, jitter (SD of latency), rise time, and decay, exhibited cell-to-cell variabilities, there were no significant differences among the groups (Supplementary Fig. 3f–j). Shorter light pulses also reliably evoked synaptic responses (Supplementary Fig. 3k). Furthermore, the paired-pulse ratio (PPR), an index of presynaptic release probability, showed no significant differences (Fig. 3f). These results suggest that adding presynaptic tags to ChR2 did not have harmful effects on its function of inducing leEPSC and on its endogenous properties of presynaptic release. Tetrodotoxin (1 μM), a voltage-dependent sodium channel blocker, completely abolished leEPSCs in all the ChR2-tag groups (Fig. 3g, h), suggesting that action potentials were elicited at presynaptic terminals by the photostimulation of ChR2, leading to synaptic transmission.

### Preferential presynaptic activation of mGluR2-PA-tagged ChR2-YFP

**Fig. 4:**
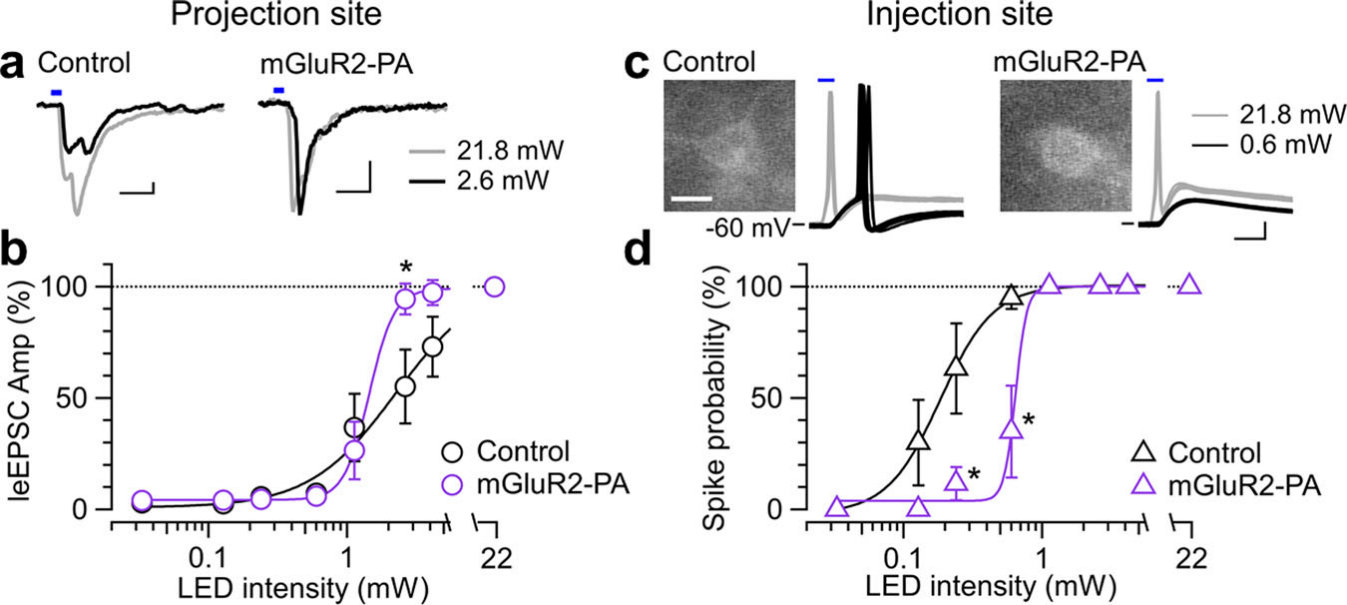
Input-output relationships of responses to photostimulation of presynaptic terminals and soma-dendrites. **a** Representative traces (average of 10 consecutive traces) of the EPSCs evoked by presynaptic stimulation with 21.8 mW (gray) and 2.6 mW (black) light intensity in the projection site in the control (left) and ChR2-mGluR2-PA (right) groups. Scale bars, 10 pA and 10 ms. **b** Input-output relationships of the light-evoked EPSCs in the control (black; *n* = 4) and ChR2-mGluR2-PA (purple; *n* = 6) groups. *P < 0.05 (two-way repeated-measures ANOVA followed by simple effects tests; interaction, *P* = 0.0118). **c** Representative traces (10 consecutive traces) of responses evoked by photostimulation of soma and dendrites with 21.8 mW (gray) and 0.6 mW (black) intensity in the injection site in the control (left) and ChR2-mGluR2-PA (right) groups. Scale bars, 10 mV and 5 ms. Insets show YFP fluorescence images of recorded cells. Scale bar, 10 μm. **d** Input-output relationships of action potentials evoked by photostimulation in the control (black; *n* = 6) and ChR2-mGluR2-PA (purple; *n* = 6) groups. *P < 0.05 (two-way repeated-measures ANOVA followed by simple effects tests; interaction, *P* = 0.0139).

Based on our results, mGluR2-PA was considered as the most prominent candidate for a presynaptic localization tag with the ChR2 function intact. Because mGluR2-PA preferentially accumulated ChR2 at presynaptic terminals, it would enable induction of synaptic responses more effectively than the control. Hence, we examined the input-output relationship of the photostimulation intensity at the CeA (projection site) and the leEPSC amplitude in CeA neurons. The leEPSC amplitude in the control was largely diminished when the photostimulation intensity was reduced to 2–4 mW, whereas it was preserved in the mGluR2-PA group (Fig. 4a, b). The amplitude at 2.6 mW intensity was significantly larger in the mGluR2-PA group than that in the control (Fig. 4b), indicating that the mGluR2-PA made the PB-CeA terminals more responsive to moderate photostimulation. In contrast, if the mGluR2-PA suppresses the ChR2 expression at the soma and dendrites, stronger photostimulation compared with the control would be required to evoke action potentials in the soma and dendrites of PB neurons. To test this, we recorded light-evoked action potentials from the soma of YFP-positive PB neurons (injection site) in the control and mGluR2-PA groups. In clearly YFP-positive neurons, maximum photostimulation intensity readily evoked action potentials in both the control and mGluR2-PA groups. However, weaker stimuli failed to evoke action potentials more frequently in the mGluR2-PA group than in the control group, and thus, the input-output curve was largely right-shifted (Fig. 4c, d). The probabilities of action potential generation at the intensities of 0.6 and 0.24 mW were significantly lower in the mGluR2-PA group than those in the control (Fig. 4d). Altogether, the mGluR2-PA tag preferentially accumulated ChR2 expression in the axon terminal regions and suppressed it in the soma and dendritic regions.

### Application of mGluR2-PA-tagged ChR2-YFP to the spike collision test

**Fig. 5:**
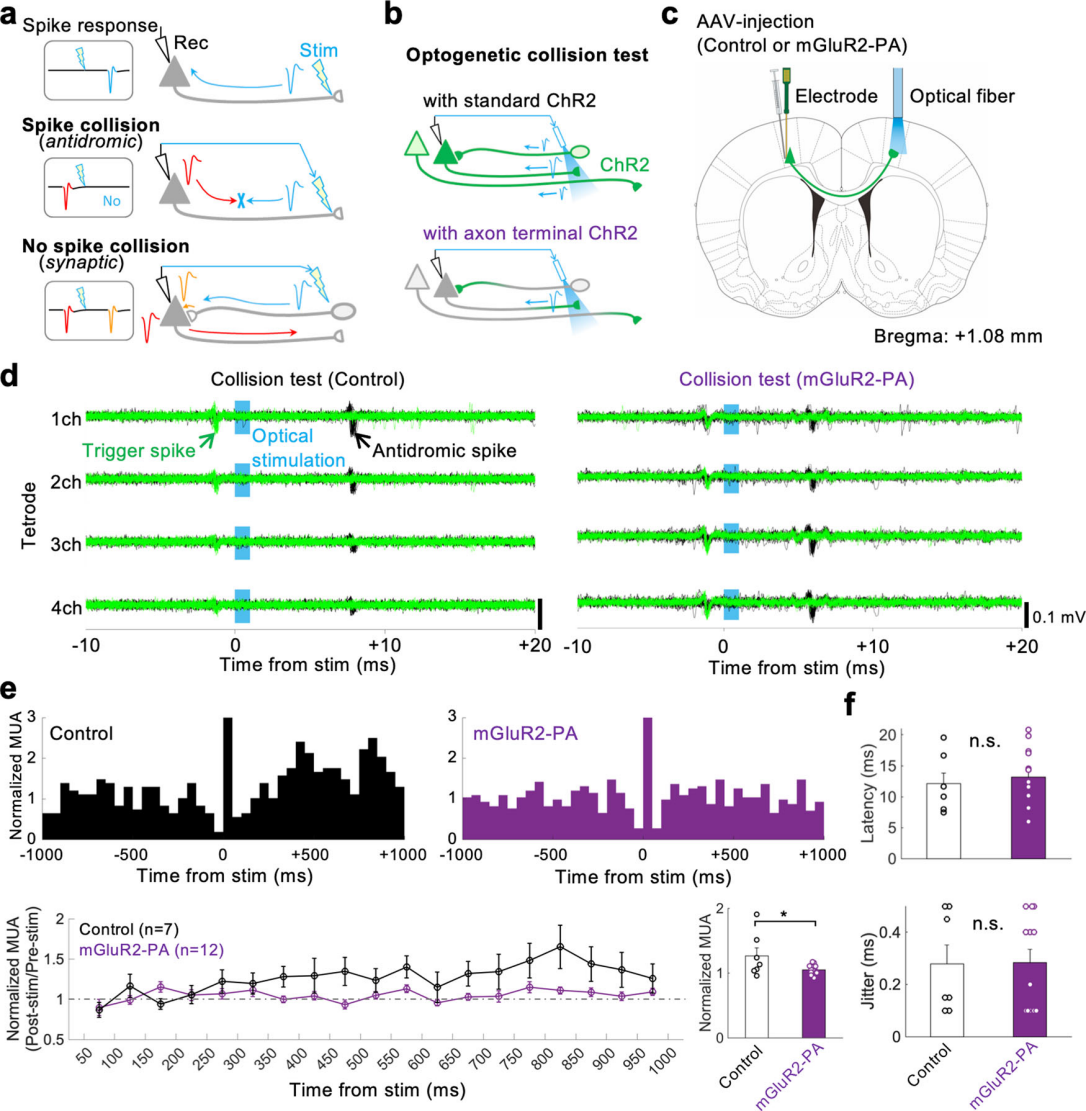
Antidromic spikes evoked by ChR2-mGluR2-PA in the spike collision test. **a** Spike collision test for identifying axonal projection (modified from ref. ^24^). An antidromically evoked spike (blue) disappears because of a collision with a spontaneously occurring spike (red) along the axon, meaning true axonal projection. Otherwise, the spike is determined as a non-antidromic (synaptic) spike (orange). **b** ChR2 localized at axon terminals improves the spatial resolution and temporal efficiency of the spike collision test. It reduces synaptic spikes by excess excitation of other neurons and antidromic spikes from axons merely passing the stimulation site. **c** Spike collision test for the callosal projection of rat cortical neurons. The AAV-DJ vector was injected into the primary motor cortex (M1) of the left hemisphere to express control ChR2 or ChR2-mGluR2-PA. In the collision test, a silicon probe for spike recording was inserted in the left M1, and an optical fiber for antidromic stimulation was set on the surface of the right M1. **d** Successful examples of spike collision test using control ChR2 (left) and ChR2-mGluR2-PA (right). Black traces represent antidromic spikes in response to photostimulation (cyan). Green traces show the disappearance of the antidromic spike in the presence of a spontaneous (trigger) spike. **e** Suppression of post-stimulus excitation of unrelated neurons by ChR2-mGluR2-PA. Upper: multiple unit activity (MUA) histograms (spike activity of multiple neurons; 50 ms bins; 0 ms, the onset of stimulation) from the same collision test data as shown in (**d**). Lower left: time course of normalized MUA following the stimulation (mean ± SEM). Lower right: comparison of normalized MUA average of 1000 ms after stimulation between the control ChR2 (*n* = 7) and ChR2-mGluR2-PA (*n* = 12). *P < 0.05, student’s *t*-test. **f** No significant difference in the latency (control ChR2: 12 ± 1.7 ms, ChR2-mGluR2-PA: 13 ± 2.5 ms; mean ± SEM) and jitter (control ChR2: 0.28 ± 0.07 ms; ChR2-mGluR2-PA: 0.28 ± 0.07 ms) of antidromic spikes between the control ChR2 and ChR2-mGluR2-PA. n.s., not significant, student’s *t*-test.

To prove the practical application of mGluR2-PA-tagged ChR2 in vivo, we performed an optogenetic spike collision test [Multi-Linc method^24^] based on an electrophysiological technique to determine axonal projection by the collision of an antidromic spike with a spontaneous spike (Fig. 5a). If the standard ChR2 is widely expressed in differently located neurons, the antidromic stimulation may cause unnecessary spikes by exciting somata, dendrites, or passing axons of unrelated neurons, which reduces the spatial resolution and temporal efficiency of the spike collision test. Therefore, preferential localization of ChR2 at axon terminals is expected to eventually improve the resolution and efficiency of the optogenetic spike collision test (Fig. 5b). Here, we tested whether the mGluR2-PA-tagged ChR2 can be used in the optogenetic spike collision test in place of control ChR2. The spike collision test was applied to contralaterally projecting pyramidal cells, which expressed mGluR2-PA-tagged ChR2, in the primary motor cortex (M1) of unanesthetized rats (Fig. 5c). Figure 5d demonstrates successful spike collision in representative neurons expressing control or mGluR2-PA-tagged ChR2; light-evoked antidromic spikes completely disappeared upon collision with spontaneous (trigger) spikes. Using the mGluR2-PA-tagged ChR2 we identified cortical projection neurons by the spike collision test at least as well as when using the control (control: 7 successes in 18 sessions in 4 rats; mGluR2-PA: 12 successes in 16 sessions in 3 rats). The antidromic spikes were evoked repetitively even at high-frequency stimulation in both the control and mGluR2-PA groups (frequency following test, Supplementary Video 1). To assess unnecessary spike noises, we analyzed multiple unit activity (MUA) changes after the photostimulation using the same tetrode traces that we used to observe the spike collision above (Fig. 5e). While a single photostimulation pulse not only evoked an antidromic spike but also increased the MUA noise over 1000 ms in the control group, the MUA increase was suppressed in the mGluR2-PA group (Fig. 5e). The excess MUA noise was significantly reduced in the mGluR2-PA group compared with the control during 1000 ms after photostimulation (Fig. 5e, lower right). The latency and jitter of antidromic spikes were not significantly different between the groups (Fig. 5f). These results indicate that our mGluR2-PA tag is practically applicable for the optogenetic spike collision test with less noise.

## Discussion

In this report, we demonstrated that the mGluR2-PA tag effectively localized ChR2 at axon terminals. The mGluR2-PA-tagged ChR2 induced synaptic transmission with weaker photostimulation in axonal terminals of long-range projection. Furthermore, it was practically applicable for the spike collision test to identify axonal projection with less polysynaptic noise. Thus, we suggest that the mGluR2-PA tag can be applied to optogenetics and other actuators and that it opens several possibilities for optogenetic experiments.

Recently, various methods and tools have been developed to localize opsins to cellular organelles and subcellular domains in cells^7^. Regarding localization to presynapses, a reduction in the photoresponse of ChR2 in dendrites has been investigated electrophysiologically by attaching the MVIBD tag to ChR2, but the pattern of expression in neurons was not examined^20^. The neurexin 1α C-terminal tag was used for presynaptic accumulation, but its functional improvement remains unclear^21,22^. The MVIBD tag used here tended to form aggregates in the cell body and was not distributed in axons (Fig. 1c). Here, we showed that addition of the mGluR2 C-terminal domain to ChR2 increased colocalization with Bassoon in cultured neurons without forming aggregates, suggesting that mGluR2 may be effective in stably localizing ChR2 to presynaptic terminals (Fig. 1f). While most mGluR subtypes have been found to bind to a variety of molecules through their respective intracellular C-terminal domains, the target molecules that bind to the C-terminal of mGluR2 are largely unknown^34^. Stowell and Craig have suggested that the mGluR2 C-terminal has a transport function to prevent it from reaching axons^35^, which may conflict with our observations (Fig. 1f). This discrepancy could be explained by the difference in neuronal maturation or vector infection. They observed the localization in cultured hippocampal neurons at an immature stage (DIV13–15) one day after gene transfer by HSV vectors, whereas we observed it in mature neurons several weeks after AAV vector infection (Fig. 1). Together with a previous report that mGluR2 starts localizing to axon terminals around postnatal day 9 in the mouse thalamus^36^, the transport mechanism of mGluR2 may change during neuronal development in cultured neurons. Moreover, an interaction of mGluR2 with postsynaptically localized Tamalin via its PDZ-binding motif has been reported in a yeast two-hybrid system and GST pull-down assays^33^, although direct binding in the brain tissue has not been confirmed. As our mGluR2-PA has no PDZ-binding motif due to the addition of the PEST sequence, we could inhibit such postsynaptic interaction and localization. Furthermore, the axon-trafficking sequence (ATE) of IMPA1^28^ would contribute to enhancing the presynaptic localization. AAV transfection sometimes causes retrograde or anterograde ChR2 expression ectopically in unintended neurons^37^. In this study, we hardly found such ectopically ChR2-expressing neurons in the projection and other sites (Figs. 2–4). The degree to which the mGluR2-PA tag affects the ectopic expression after AAV transfection remains to be evaluated.

Herein, we suggest that the mGluR2-PA tag may be useful for other actuators, contributing to neural circuit research. We expect that in the future new tags will be designed by different strategies. For example, mGluR4a and mGluR7a localize to the AZ^26^, and thus may localize ChR2 further to the terminals than mGluR2. Furthermore, although in the current study the N-terminal domain of the AZ protein, CAST, and the RIM1 PDZ domain did not work well owing to aggregation (Fig. 1b, c), i.e., folding problems, these regions are important for localization to synaptic terminals^31^. Therefore, once the amount of expression and folding is controlled, which prevents and improves aggregation and localization, these CAST and RIM1 regions could be used as effective tags for localizing actuators to AZs.

The mGluR2-PA tag is also a useful tool for studying basic synaptic transmission mechanisms in long-projecting axonal terminals. In general, combining optogenetics with electrophysiology enables us to selectively activate just the presynaptic axons of interest, which is technically impossible by classical electrophysiology using stimulation electrodes. Thus, it is an extremely useful method, especially for assessing functional connections including the long-range pathway from the brain stem to the amygdaloid, as we focused on here^38^. A problem with this method is that virally transfected ChR2 tends to be overexpressed all over the cell membranes in a spatially non-selective manner, and thus photostimulation can recruit not only the projection axons but also passing fibers expressing ChR2s, which would disrupt the spatial and/or temporal accuracy of the experimental design. The localization tag established in the present study therefore has an advantage because of its low expression in the dendrite and axonal region, which reduces contamination of the photostimulation. It should be noted that, although ChR2-mGluR2-PA decreased the somatic response and increased the terminal response to photostimulation, the weak (subthreshold) stimulation that evoked no action spikes at the soma was not enough to evoke synaptic transmission at the terminals in our experimental condition (Fig. 4b and d). It would be a challenging but important future goal to improve the presynaptic tags to more selectively stimulate only presynaptic terminals. Another advantage of the mGluR-PA tag is that it does not appear to distort endogenous vesicular release. While several opsins have been targeted to presynaptic terminals by fusion with synaptic vesicle proteins^7^, their functional effects on vesicular filling and/or dynamics have not been fully elucidated. We attempted to localize ChR2 not too close to the release sites, yet enrich it at the perisynaptic regions. To fulfill these ambivalent objectives, we used the C-terminal domain of mGluR2, whose expression is highly enriched in the perisynaptic region as discussed above, and indeed our electrophysiological analysis revealed no functional distortion of the release probabilities or the synaptic responses (Fig. 3 and Supplementary Fig. 3). It should be noted, however, that we cannot confirm whether this synaptic transmission is physiological, i.e., as it would appear in vivo. The expression properties of mGluR-PA-tagged ChR2 provide a useful tool for studying the neuronal circuit of long-projection pathways in the future.

The optogenetic spike collision test^23–25,39,40^ is a powerful method for identifying interareal axonal projections of recorded neurons. Unlike electrical stimulation, optogenetic stimulation can efficiently activate axons in a cell-type- or pathway-specific manner with no electrolytic damage. We typically use viral vectors or genetically modified animals to express ChR2 in target neurons. Viral vector infection often results in sparse and heterogeneous ChR2 expression in only a small number of neurons at the local injection site, whereas single or crossed transgenic animals enable homogeneous ChR2 expression in the same type of neurons in the entire brain^24^. Therefore, the use of transgenic animals is more advantageous for optimizing the optogenetic spike collision test in a given target area. However, such multi-areal ChR2 expression in transgenic animals has the limitation that optical stimulation could activate not only the target axons but also somata, dendrites, or passing axons of unrelated neurons around there. These unfavorable activations may evoke spike noise, and thus reduce the resolution and efficiency of the spike collision test. This problem can be solved by presynapse tags like mGluR2-PA that localize ChR2 at axon terminals. Even with AAV vector infection, we successfully demonstrated the optogenetic spike collision test for identifying long-range axons from cortical neurons expressing mGluR2-PA-tagged ChR2 with the property of antidromic spike remaining intact. Moreover, we confirmed the reduction in spike noise derived from other neurons. These results encourage us to introduce mGluR2-PA-tagged ChR2 to genetically modified animals to improve the resolution and efficiency of the spike collision test.

Now that we can evoke synaptic releases and antidromic spikes in vitro and in vivo, our mGluR2-PA-tagged ChR2 will be a useful research tool in various fields of neuroscience at the molecular to circuit levels.

## Methods

### Ethics

All recombinant DNA and animal experiments in this study were performed following regulations and guidelines for the care and use of experimental animals and approved by the Institutional Committee for the Care and Use of Experimental Animals at the University of Yamanashi (protocol #A30-21), by the Institutional Animal Care and Use Committee of the Jikei University (protocol #2017-048), and by the Institutional Animal Care and Use Committee of Tokyo Medical and Dental University (protocol #A2019-274). All experiments conformed to the Guidelines for the Proper Conduct of Animal Experiments of the Science Council of Japan (2006).

### AAV vector preparation and production

The ChR2-expressing AAV vector, named ChR2-YFP, was modified from pAAV-hSyn-hChR2(H134R)-EYFP [a gift from Karl Deisseroth (Addgene plasmid #26973)], in which restriction enzyme sites in non-coding areas and non-recognized sequences in the protein-encoding region were removed. The following DNA was synthesized and subcloned into the MCS, which was inserted after the EYFP: mouse mGluR2 C-terminal (820–872 aa from NCBI entry NM_001160353), ATE (rat *IMPA1* 1126–1249 nt from GU441530), and PEST (mouse VEGFR2 1171–1209 aa from NM_010612). MVIBD (Optineurin 420–524 aa from NM_001356487 and DAB2 429–516 aa from NM_023118) was cloned from mouse brain cDNA. Mouse CAST (139–306 aa) and human RIM1 (614–693 aa) have previously been described^31,41^.

The AAV vector solution was prepared by transfecting pAAV-hSyn-ChR2-YFP with tags, pAAV-DJ, and pHelper into AAV293 cells using PEI-Max (Polysciences, Inc., Warrington, PA, USA). After 4 days, cells were centrifuged at 1600 × *g* for 5 min and resuspended with 150 µL of phosphate-buffered saline (PBS). Cells were lyzed by freezing and thawing four times using liquid N_2_, followed by incubation in benzonase at 45 °C for 15 min. The crude lysates were centrifuged at 21,500 × *g* for 10 min at 4 °C and the vector-containing supernatants were collected. This step was repeated three times. The AAV titers were confirmed by RT-PCR and stored at −80 °C.

### Evaluation of AAV expression in primary hippocampal neuronal culture

The preparation of primary rat hippocampal neurons has previously been described^42^. Briefly, hippocampi were dissected from Wistar rat embryonic day 18 brains and dissociated by papain at 37 °C. The neurons were plated on poly-D-lysine (PDL)-coated 24-well plates at 50,000 cells/well for immunoblotting, and at 3000 cells/well for immunocytochemistry. Cultures were maintained in a Neurobasal medium containing B-27 supplement and GlutaMAX (Gibco, Carlsbad, CA, USA) and kept in an incubator with 5% CO_2_ at 37 °C. Cells were infected with AAVs after 10–13 days in vitro (DIV) and left for at least 10 days before preparation for immunoblotting and immunostaining.

For western blotting, the cells were washed with PBS, extracted with lysis buffer [20 mM Tris-HCl, pH 7.5, 150 mM NaCl, 0.5 mM EDTA, 1% Triton X-100, and protease inhibitor cOmplete (Roche Diagnostics, Tokyo, Japan)], and boiled with SDS sample buffer. The samples were applied to SDS-PAGE and transferred to a PVDF membrane. The membrane was blocked with 5% skim milk in tris-buffered saline with Tween 20 (TBST) for 1 h, incubated with anti-GFP antibody (Invitrogen, Carlsbad, CA, USA) for 1 h, followed by HRP-conjugated secondary antibody for 1 h, with TBST washes between incubations. Chemical luminescence using Immobilon Forte (Millipore, Darmstadt, Germany) was detected by LAS4000 mini (GE Healthcare Life Sciences, Chicago, IL, USA). Bands’ intensity was measured by the expand ImageJ version Fiji.

For immunostaining, the cells were washed with PBS and fixed with 4% paraformaldehyde in PBS for 30 min. After washing with PBS, cells were permeabilized with 0.25% Triton X-100 in PBS for 15 min and blocked with 1% BSA and 0.25% Triton X-100 in PBS for 30 min. Cells were incubated with anti-Tau (Synaptic System, Goettingen, Germany), anti-MAP2 (Sigma-Aldrich, St. Louis, MO, USA), or anti-Bassoon (ENZO Life Sciences, New York, NY, USA) antibodies diluted in blocking buffer for 2 h at room temperature (RT), followed by Alexa-Fluor-conjugated secondary antibodies. Images of stained cells were captured with confocal laser microscopy (FV1200, Olympus, Tokyo, Japan) equipped with a ×60 oil immersion objective lens (UPLSAPO60X, NA 1.35; Olympus) with confocal aperture 300. Pearson’s R-value between ChR2-YFP and subcellular markers was calculated by the coloc2 plug-in of Fiji. Images were background-subtracted using a 15-micron rolling ball algorithm and a threshold regression-selected Bisection.

### AAV injection into the hippocampus and cortex for in vivo anatomical studies

To examine the contralateral projection of pyramidal neurons in the hippocampus and cortex, adult C57BL/6 mice (Charles River, Wilmington, MA, USA) were anesthetized with a Ketamine (130 mg/kg, i.p.) and Xylazine (13 mg/kg, i.p.) mixture and placed on a mouse stereotactic frame (Stoelting, Wood Dale, IL, USA). A small craniotomy was performed over the cortex with a drill, and AAV vector solution (1–1.4 µL), AAV-DJ-Syn-ChR2-YFP (provided by Dr. Kenta Kobayashi, 3.0 × 10^10^ vg/µL) and AAV-DJ-Syn-ChR2-YFP-mGluR2-PA (from Dr. Kenta Kobayashi, 2.3 × 10^10^ vg/µL) were stereotactically injected into the hippocampal CA3 area (stereotactic coordinates: 2.8 mm to lateral, 2.3 mm to posterior from Bregma, and 1.8 mm deep from brain surface) and the motor cortex (stereotactic coordinates: 1.5 mm to lateral, 1.1 mm to anterior from Bregma, and 0.7 mm deep from brain surface) through a glass micropipette attached to a Nanoject III (Drummond, Broomall, PA, USA) at a rate of 10–20 nL/s.

Two to three weeks after the surgery, virus-injected mice were deeply anesthetized again with pentobarbital and perfused transcardially with 10–20 mL of PBS (pH 7.4) first, followed by 70 mL of 4% paraformaldehyde and 10% picric acid in 0.1 M PB (pH 7.4). Brains were removed and cut into 100 -µm-thick coronal sections at the hippocampus or prefrontal cortex using a Microslicer (DTK-1000N, Dosaka, Kyoto, Japan). Sections were counterstained with DAPI for nuclear labeling and mounted in Vectashield (Vector Lab, Burlingame, CA, USA). The EYFP fluorescent signal was imaged with a confocal microscope (FV1000, Olympus). The contralateral and ipsilateral areas were observed with a ×20 objective lens (UPLSPO 20X, NA 0.75; Olympus) with the same setting for each section. For the hippocampus, the average fluorescence intensity at the three points of region-of-interest (ROI, 62 × 62 µm) was quantified for the ipsilateral pyramidal cell layer and contralateral stratum oriens. For the cortex, the average fluorescence intensity at the three points of ROI (124 × 124 µm) was quantified.

### Pre-embedding immunoelectron microscopy

For pre-embedding immunogold labeling, three weeks after the AAV injection to the hippocampal CA3 area, mice were deeply anesthetized using pentobarbital and perfused transcardially with PBS, followed by 4% paraformaldehyde and 10% picric acid in 0.1 M PB (pH 7.4). The 100-µm-thick coronal sections were cut at the midline to divide the ipsilateral and contralateral hippocampal slices. Then, sections were incubated with 30% sucrose in 0.1 M PB for at least 2 h for cryoprotection and dipped in liquid N_2_ for 1 min. After the freeze-thaw step, sections were washed with TBS, blocked with 20% normal goat serum (NGS) in TBS for 30 min, and incubated overnight with anti-GFP antibody (Invitrogen) in TBS containing 2% NGS. After washing, sections were incubated overnight with 1.4 nm gold-coupled anti-rabbit IgG (Nanoprobes), and then postfixed with 1% glutaraldehyde for 10 min before silver enhancement with the HQ Silver Kit (Nanoprobes, Yaphank, NY, USA). Then, immunolabeled sections were treated with 1% OsO_4_ in 0.1 M PB for 40 min, counterstained with 1% uranyl acetate for 35 min, dehydrated, and flat embedded in Durcupan resin (Sigma-Aldrich) at 60 °C for 48 h. Ultrathin sections were then prepared by Ultramicrotome EM UC7 (Leica, Wetzlar, Germany) and examined with a transmission electron microscope (JEM2100F, JEOL, Tokyo, Japan).

In three individual mouse samples, the contralateral CA1 area was targeted. We collected images containing fine structures with more than five gold particles. For the control group, 291, 183, and 247 fine structures from 108, 88, and 104 images, respectively, and for the mGluR2-PA group, 126, 223, and 99 fine structures from 56, 160, and 62 images, respectively, were classified into terminal, which has more than five synaptic vesicles, AZ, which has labeling on the synapse, and other for the axon.

### Surgery for AAV infection of mice for electrophysiology in acute brain slices

For the AAV injection, male C57BL/6J mice (4 weeks old; Japan SLC, Shizuoka, Japan) were anesthetized with a mixture of medetomidine hydrochloride (0.75 mg/kg; Zenoaq, Fukushima, Japan), midazolam (4.0 mg/kg; Astellas, Tokyo, Japan), and butorphanol tartrate (5.0 mg/kg; Meiji Seika Pharma, Tokyo, Japan) and placed on a stereotaxic instrument (SR-6M-HT, Narishige, Tokyo, Japan). AAV vector solution, AAV-DJ-Syn-ChR2-YFP, -mGluR2, -MVIBD, -CAST, -RIM1, or -mGluR2-PA (250 nL; 2.9 × 10^7^–2.3 × 10^10^ vg/μL) was injected into the bilateral parabrachial nuclei (1.5 mm to lateral, 6.0 mm to posterior from Bregma, and 3.4 mm deep with a 20° anterior to posterior angle) using a Hamilton microsyringe (1701RN Neuros Syringe, 33 G, 10 μL, Hamilton, Reno, NV, USA) at an injection speed of 50 nL/min controlled with a microinjection syringe pump (UMP3, World Precision Instruments, Sarasota, FL, USA) as previously reported^38^. The injection syringes were left in place for 10 min before withdrawal. In the control group, AAV-DJ-Syn-ChR2-YFP was diluted with PBS 50–100 times to adjust the expression level of ChR2-YFP in the projection site to that of the variants with presynaptic-localizing tags.

### Whole-cell electrophysiology and photostimulation of acute brain slices

Five to six weeks after AAV injection, the mice were deeply anesthetized with isoflurane (5% in 100% O_2_) and decapitated. The brains were quickly removed into an ice-cold cutting solution consisting of (in mM) 92 *N*-Methyl-D-glucamine, 2.5 KCl, 0.5 CaCl_2_, 10 MgSO_4_, 1.25 NaH_2_PO_4_, 2 thiourea, 3 sodium pyruvate, 12 *N*-acetyl-L-cysteine, 25 D-glucose, 5 L-ascorbic acid, 20 HEPES, and 30 NaHCO_3_, equilibrated with 95% O_2_ + 5% CO_2_ (pH ~7.4; osmolality, ~290 mOsm/kg). Coronal slices containing the amygdala or parabrachial nucleus were cut with a vibrating blade slicer (VT1200S, Leica). The slices were first incubated in the cutting solution at ~34 °C for 10–15 min, and then kept at RT (20–25 °C) in standard artificial cerebrospinal fluid (ACSF) consisting of (in mM) 125 NaCl, 3 KCl, 2 CaCl_2_, 1.3 MgCl_2_, 1.25 NaH_2_PO_4_, 10 D-glucose, 0.4 L-ascorbic acid, and 25 NaHCO_3_ (pH ~7.4 bubbled with 95% O_2_ + 5% CO_2_; osmolality, ~300 mOsm/kg) until whole-cell recordings. A slice was transferred to a recording chamber (RC-26GS, Warner Instruments, Holliston, MA, USA) and continuously superfused at a rate of 1.5–2.5 mL/min with standard ACSF at 30–32 °C.

Whole-cell patch-clamp recordings were conducted on central nucleus of the amygdala (CeA) and parabrachial nucleus (PB) neurons, which were visually identified under an upright microscope with oblique illumination (BX-51WI, Olympus) as previously reported^43^. YFP fluorescence was briefly examined before the recordings to confirm ChR2 expression in the CeA and to explore YFP-positive cells in the PB. YFP-positive cells were not found in the CeA in which some neurons project to the PB. Oblique illumination and epifluorescence images were captured using a CCD camera (IR-1000, DAGE-MTI, Michigan City, IN, USA). A patch-clamp electrode (4–8 MΩ) made of borosilicate glass pipettes (1B150F-4, World Precision Instruments) was filled with internal solution composed of (in mM) 122.5 potassium gluconate, 10 HEPES, 17.5 KCl, 0.2 EGTA, 8 NaCl, 2 MgATP, and 0.3 NaGTP (pH 7.2; osmolarity, 290–300 mOsm). To isolate leEPSCs at the CeA, cells were held at −60 mV and recorded in the presence of picrotoxin (100 μM).

For the recordings of light-evoked responses in the injection site (PB), the membrane potential was recorded from YFP-expressing cells in the presence of kynurenic acid (3 mM) and picrotoxin to block synaptic inputs. A continuous current was injected manually to keep the membrane potential at ~−60 mV. The membrane current and membrane potential were recorded with a MultiClamp 700B amplifier (Molecular Devices, San Jose, CA, USA), filtered at 2 kHz, and digitized at 10 kHz with a 16-bit resolution using a PowerLab interface (AD Instruments, Colorado Springs, CO, USA), together with timing pulses for light stimulation. The ChR2 was activated using an LED illumination system mounted on the microscope (470 nm; M470L3, Thorlabs, Newton, NJ, USA), delivered to the entire field through a ×40 water-immersion objective lens (LUMPLFLN40XW, NA 0.8; Olympus). Photostimulation timing and duration were controlled by Master-8 (A.M.P.I., Jerusalem, Israel), and the intensity was controlled using an LED driver (LEDD1B, Thorlabs) and measured using a digital optical power meter (9742-10/3664; Hioki, Nagano, Japan). Light stimuli were applied every 20 s. Paired-pulse ratio (PPR) was defined as the ratio of the second EPSC amplitude to the first EPSC amplitude, in response to two stimuli with a 100-ms interstimulus interval.

The recorded membrane currents and membrane potentials were analyzed offline with Igor Pro 7 (WaveMetrics, Portland, OR, USA). The fluorescence intensity in the CeA, especially the capsular part, was measured in the epifluorescence images and the intensity in the basolateral amygdala (BLA), which receives no projection from the PB, was subtracted as the background using ImageJ.

### Surgery for AAV infection for rat electrophysiological recordings

Adult Long-Evans rats were anesthetized with isoflurane (4.5% for induction and 2.0–2.5% for maintenance; body temperature 37 °C) on a stereotaxic frame (SR-10R-HT, Narishige). AAV vector solution (AAV-DJ-Syn-hChR2-YFP, 1.8 × 10^10^ vg/mL; AAV-DJ-Syn-hChR2-YFP-mGluR2-PEST-ATE, 2.3 × 10^10^ vg/mL, 1.0 µL) was injected slowly into the primary motor cortex of the left hemisphere (2.5 mm to lateral, 1.0 mm to anterior from Bregma, and 1.0 mm deep from brain surface) using a microsyringe pump (Legato100, Kd Scientific, Holliston, MA, USA).

After the AAV injection, reference and ground electrodes were implanted above the cerebellum, a head plate (CFR-2, Narishige) was attached to the skull with small anchor screws and dental resin cement. The exposed surface of the skull and brain was covered with silicone sealant. Analgesics and antibiotics were applied postoperatively as required (meloxicam, 1 mg/kg s.c.; 0.1% gentamicin ointment, *ad usum externum*).

### In vivo electrophysiological recordings with photostimulation

Three weeks after the surgery for AAV injection, we performed extracellular multichannel recordings through a 32-channel silicon probe (a32-Isomura-6-14-r2-A32 or ISO-3x-tet-A32, NeuroNexus Technologies, Ann Arbor, MI, USA) from the injection site of the left M1 area (1200 µm, putative layer 5) under head-fixation without anesthesia. The extracellular signals were amplified (final gain ×2000) and filtered (0.5 Hz to 10 kHz) through a 32-channel head-stage (MPA32I, Multi Channel Systems) and main amplifier (FA64I, Multi Channel Systems, Reutlingen, Germany). The signals were digitized at 20 kHz and 12 bit and recorded with a 32-channel hard-disc recorder (LX-120, TEAC, Tokyo, Japan). These signals included spike activity of multiple neurons and local field potentials (LFPs). The track of the silicon probe and the expression of ChR2-EYFP were confirmed later by fluorescence microscopy histologically.

During the rat in vivo electrophysiology, the contralateral (right) M1 area was photostimulated to evoke antidromic spikes at axon terminals for the spike collision test as previously reported [Multi-Linc analysis^24,39,40^]. An optical fiber (FT400EMT, FC, Thorlabs; core diameter 400 μm) was placed on the surface of the right M1 area, where intratelencephalic (IT) pyramidal cells of the left M1 area should innervate with axon terminals with ChR2. A plus of blue light (intensity 15 mW; duration 1.0 ms) was applied through the optical fiber using an ultra-high-power LED light source (UHP-Mic-LED-460, Prizmatix, Holon, Israel) and a stimulator (SEN-8203, Nihon Kohden, Tokyo, Japan). We performed a spike collision test by evoking the antidromic spike immediately (0–1.0 ms) after detecting a spontaneous spike with a similar spike-waveform. The success of spike collision was determined in post hoc analysis as follows.

### Spike collision analysis

The raw signal data were processed offline to isolate spike events of individual neurons in each tetrode of the silicon probe, using the semiautomatic spike-clustering software, EToS, and the manual cluster-refining software, Klusters, with NeuroScope. In the spike collision analysis, we tested whether a possible antidromic spike disappeared by the precedence of a spontaneous spike of the same spike cluster^24^. We counted the occurrence of an antidromic spike in the presence (test trials) and absence (control trials) of a preceding spike, and statistically justified it by a 2 × 2 *χ*^2^ test for spike and no-spike counts in the control and test trials. See previous reports for details on the spike collision test^24,39,40^.

### Statistics and reproducibility

Data in the text and figures are expressed as mean ± SEM of the sample number (*n*). The sample sizes were indicated in the figure legends. We used appropriate statistical tests with post hoc analyses when applicable, i.e., Student’s or Welch’s *t*-test, one-way ANOVA with Tukey’s or Bonferroni’s multiple comparison tests, two-way repeated-measures ANOVA with simple effects tests, Kruskal–Wallis test with Steel–Dwass test, and χ^2^ tests with residual analysis. The differences were considered statistically significant when *P* < 0.05. See “Results” for details.

### Reporting summary

Further information on research design is available in the Nature Research Reporting Summary linked to this article.

## Supplementary information

Supplementary Information

Description of Additional Supplementary Files

Supplementary Movie 1

Supplementary Data 1

Reporting Summary

## Data Availability

All source data for graphs can be found in Supplementary Data 1. Other data are available from the corresponding author on reasonable request.
