# Supplementary material for: An engineered channelrhodopsin optimized for axon terminal activation and circuit mapping
Source: Commun Biol. 2021 Apr 12;4:461. doi: 10.1038/s42003-021-01977-7 (PMC8042110; doi:10.1038/s42003-021-01977-7)
Supplement: Supplementary file 1 — Supplementary Information [file 42003_2021_1977_MOESM1_ESM.pdf]

a

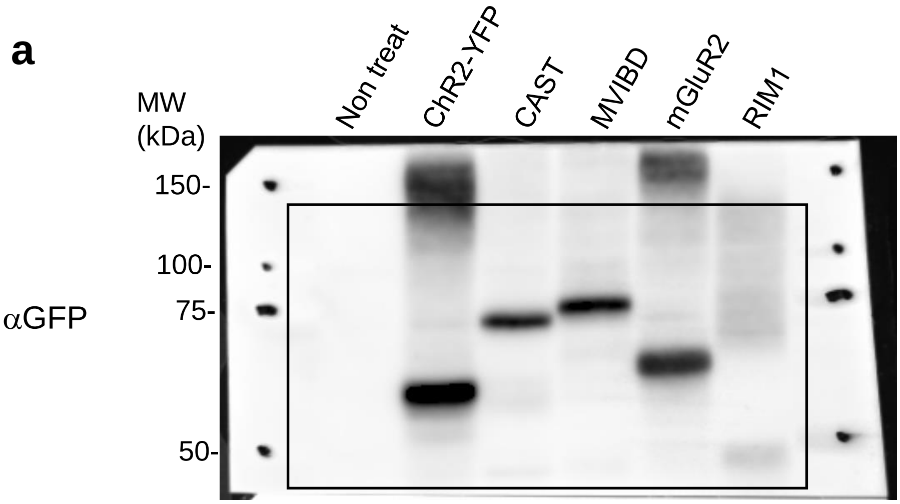

b

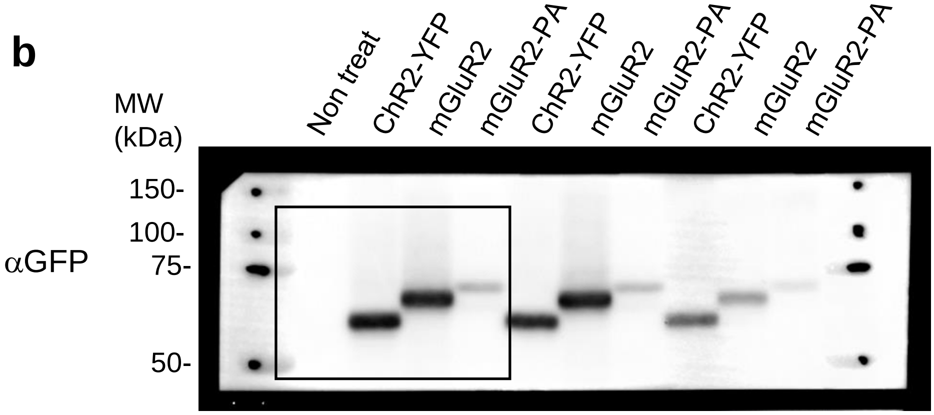

Supplementary Fig. 1. Uncropped western blot images of Figure 1

(a) Uncropped image of Figure 1b. **(b)** Uncropped image of Figure 1d.

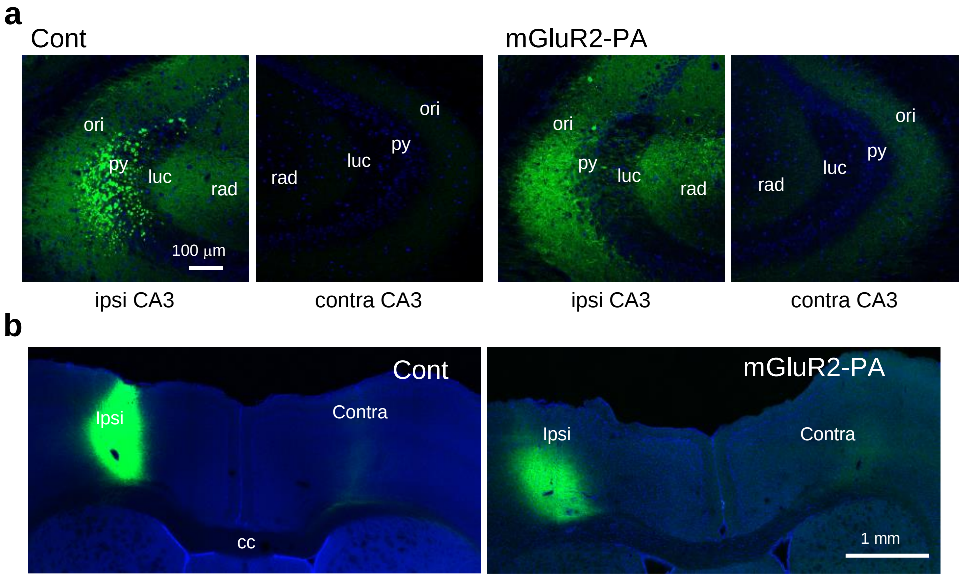

Supplementary Fig. 2. Expression of ChR2-YFP for the contralateral projection terminal in the hippocampus and cortex

(a) Representative images of hippocampal expression of ChR2-YFP. The control showed a bright signal at the pyramidal cell layer of ipsilateral CA3, whereas mGluR2-PA suppressed the ChR2-YFP fluorescence at the soma, which improved the relative fluorescence at the contralateral hippocampus (contra, CA3). **(b)** Similarly to the hippocampal expression of ChR2-YFP, the control and mGluR2-PA showed a bright signal at the injection site of the prefrontal cortex (ipsi), which projected to the contralateral site.

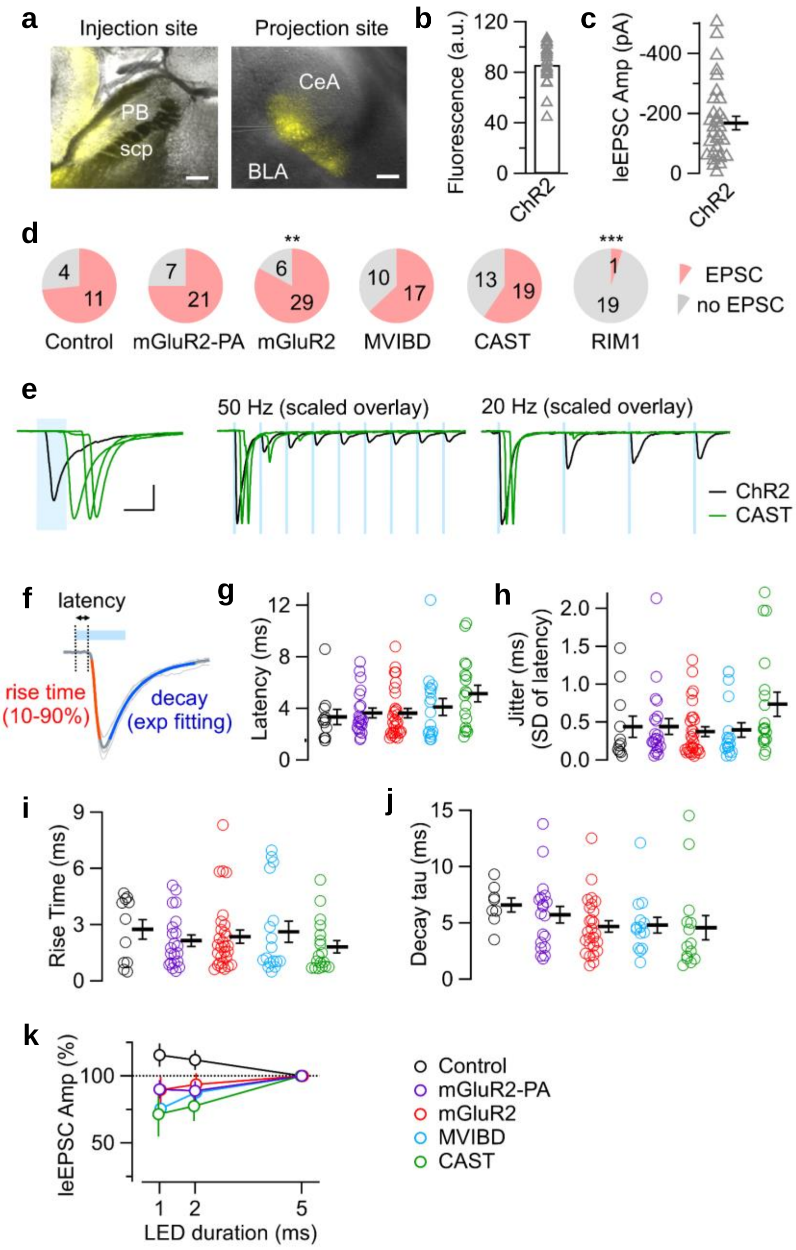

Supplementary Fig. 3. The experimental approach of whole-cell recordings in acute slices and leEPSC properties

(a) Representative images of bright field with oblique illumination merged with ChR2-YFP fluorescent images of original ChR2 in the PB, the injection site (left), and the CeA, the projection site (right). **(b)** YFP fluorescence intensities of original ChR2 in the CeA (n = 20). **(c)** Summarized amplitude of leEPSCs in the original ChR2 group (n = 32). **(d)** Proportion of recorded cells with leEPSC (red, with leEPSC; gray, without leEPSC). The numbers in the pie charts indicate the number of recorded cells. *** $P < 0.001$, ** $P < 0.01$ (χ^2 test followed by residual analysis). **(e)** The traces of untypical large EPSC in the CAST group, which had a long latency (left) and/or low reliability to high frequent photostimulation (center and right) **(f)** A schematic of the quantification

of leEPSC kinetics. **(g–j)** Summary of the latency (g), jitter (SD of latency; h), rise time (10%–90% amplitude; i), and decay time constant calculated by single exponential curve fitting (traces that were not fitted by the exponential model were excludes; j) of leEPSC amplitudes. There were no significant differences (latency: $P = 0.1304$, ANOVA; control, $n = 11$, mGluR2-PA, $n = 21$, mGluR2, $n = 29$, MVIBD, $n = 17$, CAST, $n = 18$; jitter: $P = 0.1222$, ANOVA; control, $n = 11$, mGluR2-PA, $n = 21$, mGluR2, $n = 29$, MVIBD, $n = 17$, CAST, $n = 18$; rise time: $P = 0.5983$, ANOVA; control, $n = 11$, mGluR2-PA, $n = 21$, mGluR2, $n = 29$, MVIBD, $n = 17$, CAST, $n = 18$; decay: $P = 0.4085$, ANOVA; control, $n = 8$, mGluR2-PA, $n = 19$, mGluR2, $n = 26$, MVIBD, $n = 14$, CAST, $n = 14$). **(k)** Effects of duration of photostimulation on leEPSC amplitude (control, $n = 8$, mGluR2-PA, $n = 12$, mGluR2, $n = 18$, MVIBD, $n = 14$, CAST, $n = 12$). In the control group, amplitude tented to be slightly increased as the duration shortened, which might be due to calcium entering the terminals through ChR2 and/or VDCC affecting the vesicular release properties and/or ChR2 desensitization resulting less ChR2 recruitment as photostimulation duration prolonged.
