# Supplementary material for: An engineered channelrhodopsin optimized for axon terminal activation and circuit mapping
Source: Commun Biol. 2021 Apr 12;4:461. doi: 10.1038/s42003-021-01977-7 (PMC8042110; doi:10.1038/s42003-021-01977-7)
Supplement: Supplementary file 2 — Description of Additional Supplementary Files [file 42003_2021_1977_MOESM2_ESM.pdf]

Description of Additional Supplementary Files

File name: Supplementary Movie 1

Description: Demonstration of spike collision test using ChR2- mGluR2-PA.

1) Antidromic spikes evoked by photostimulation.

2) Spike collision.

3) Frequency-following test. Two-pulse stimulation at 100, 125, and 133 Hz.

File name: Supplementary Data 1

Description: Raw source data of all graphs. Each data indicates individual samples.
