# Supplementary material for: An engineered channelrhodopsin optimized for axon terminal activation and circuit mapping
Source: Commun Biol. 2021 Apr 12;4:461. doi: 10.1038/s42003-021-01977-7 (PMC8042110; doi:10.1038/s42003-021-01977-7)
Supplement: Supplementary file 5 — Reporting Summary [file 42003_2021_1977_MOESM5_ESM.pdf]

nature research

Corresponding author(s): COMMSBIO-20-3168A

Last updated by author(s): 2021-1-20

Reporting Summary

Nature Research wishes to improve the reproducibility of the work that we publish. This form provides structure for consistency and transparency in reporting. For further information on Nature Research policies, see our Editorial Policies and the Editorial Policy Checklist.

Statistics

For all statistical analyses, confirm that the following items are present in the figure legend, table legend, main text, or Methods section.

n/a	Confirmed	
☐	☒	The exact sample size (n) for each experimental group/condition, given as a discrete number and unit of measurement
☐	☒	A statement on whether measurements were taken from distinct samples or whether the same sample was measured repeatedly
☐	☒	The statistical test(s) used AND whether they are one- or two-sided<br>*Only common tests should be described solely by name; describe more complex techniques in the Methods section.*
☐	☒	A description of all covariates tested
☐	☒	A description of any assumptions or corrections, such as tests of normality and adjustment for multiple comparisons
☐	☒	A full description of the statistical parameters including central tendency (e.g. means) or other basic estimates (e.g. regression coefficient) AND variation (e.g. standard deviation) or associated estimates of uncertainty (e.g. confidence intervals)
☐	☒	For null hypothesis testing, the test statistic (e.g. F, t, r) with confidence intervals, effect sizes, degrees of freedom and P value noted<br>*Give P values as exact values whenever suitable.*
☒	☐	For Bayesian analysis, information on the choice of priors and Markov chain Monte Carlo settings
☒	☐	For hierarchical and complex designs, identification of the appropriate level for tests and full reporting of outcomes
☐	☒	Estimates of effect sizes (e.g. Cohen's d, Pearson's r), indicating how they were calculated

Our web collection on statistics for biologists contains articles on many of the points above.

Software and code

Policy information about availability of computer code

Data collection	Fig1: The band intensities of Western blots were measured by using the expand ImageJ version Fiji (NIH).<br>Fig3, 4, Supplementary Fig2: Electrophysiological data in slices was collected using LabChart 7 (AD Instruments).<br>Fig5: Spike data was collected using LX Navi (Version 3.28, TEAC).
Data analysis	Fig1: Pearson's R values between AZ tag fused ChR2 and synaptic markers were calculated by using the expand ImageJ version Fiji. Statistics were performed by Prism 6 (GraphPad).<br>Fig2: Statistics was performed by Excel (Microsoft).<br>Fig3, 4, Supplementary Fig2: Electrophysiological data in slices was analyzed using Igor Pro 7 (WaveMetrics) and statistical calculations were made with R version 2.8.1.<br>Fig5: Spike sorting was done using EToS (Takekawa et al., 2010, http://etos.sourceforge.net) and Klusters (Hazan et al., 2006, http://neurosuite.sourceforge.net). Other analyses were done using custom Matlab code (Matlab R2018a, Mathworks Inc).

For manuscripts utilizing custom algorithms or software that are central to the research but not yet described in published literature, software must be made available to editors and reviewers. We strongly encourage code deposition in a community repository (e.g. GitHub). See the Nature Research guidelines for submitting code & software for further information.

Data

Policy information about availability of data

All manuscripts must include a data availability statement. This statement should provide the following information, where applicable:

- Accession codes, unique identifiers, or web links for publicly available datasets
- A list of figures that have associated raw data
- A description of any restrictions on data availability

Neuronal data from all experiments are available on reasonable request.

Field-specific reporting

Please select the one below that is the best fit for your research. If you are not sure, read the appropriate sections before making your selection.

☒ Life sciences ☐ Behavioural & social sciences ☐ Ecological, evolutionary & environmental sciences

For a reference copy of the document with all sections, see nature.com/documents/nr-reporting-summary-flat.pdf

Life sciences study design

All studies must disclose on these points even when the disclosure is negative.

Sample size	Fig1: For western blot and immunostaining, Sample size were in the range estimated By Power Analysis.<br>Fig2, Supple Fig. 1 (in vivo expression analysis); For fluorescent intensity and electron microscopy analysis, we used 3 mice for each AAV injection, and took two different plane sections. They were in the range estimated by Power Analysis, and we could eliminate waste of animals.<br>Fig3, 4, Supplementary Fig2: Sample sizes were based on previous studies (Watabe et al, 2016).<br>Fig5: No sample size precalculation was performed.
Data exclusions	Fig1 (Immunostaining): Neurons with lesions were excluded. Image data was rejected when coloc2 program indicated unsuitable for pearson correlation.<br>Fig2, Supple Fig. 1 (in vivo expression analysis); When the AAV injected mice showed less GFP expression at the CA3 or M1 injection site, the mice were excluded from the data analysis.<br>Fig3, 4, Supplementary Fig2: Neurons showing a membrane potential more positive than −40 mV or without overshooting voltage responses were excluded.<br>Fig5: We excluded single units that were not stable over the course of the recording session in in vivo electrophysiology.
Replication	The AAV injection and expression was performed on several types of experiment including primary culture neuron and in vivo recording at different facilities, and the expression was verified at all neural networks and reproduced from western blotting to the in vivo recording.
Randomization	We did not need to randomize recorded neurons across animal groups in in vivo electrophysiology.
Blinding	Fig1 (Immunostaining): In the immunostaining, imaging were performed under the blind condition.

Reporting for specific materials, systems and methods

We require information from authors about some types of materials, experimental systems and methods used in many studies. Here, indicate whether each material, system or method listed is relevant to your study. If you are not sure if a list item applies to your research, read the appropriate section before selecting a response.

Materials & experimental systems

n/a	Involved in the study	
☐	☒	Antibodies
☐	☒	Eukaryotic cell lines
☒	☐	Palaeontology and archaeology
☐	☒	Animals and other organisms
☒	☐	Human research participants
☒	☐	Clinical data
☒	☐	Dual use research of concern

Methods

n/a	Involved in the study	
☒	☐	ChIP-seq
☒	☐	Flow cytometry
☒	☐	MRI-based neuroimaging

Antibodies

Antibodies used	We described information about antibodies used in this study in ONLINE METHODS.<br>For western blotting, anti-GFP (Invitrogen, A11122; RRID: AB_221569), and HRP-conjugated secondary antibody (Jackson:

	111-035-003; RRID: AB_2313567) were used. For immunostaining of primary neurons, primary antibodies, anti-Tau (Synaptic System, 314003; RRID: AB_993039), anti-MAP2 (Sigma, M1406; RRID: AB_477171), anti-Bassoon (ENZO Life Sciences, CAM-PS003; RRID: AB_10618753), and Alexa-Fluor conjugated secondary antibodies (Invitrogen, A11004; RRID: AB_2534072, Invitrogen: A21070; RRID: AB_2535731) were used. For immunoelectron microscopy, anti-GFP antibody (Invitrogen, A11122) and 1.4 nm gold-coupled anti-rabbit IgG (Nanoprobes: 2003; RRID: 2687591) were used.
Validation	The specificity of all primary antibodies were validated from each manufacture sites with references

Eukaryotic cell lines

Policy information about cell lines

Cell line source(s)	AAV293 which was derived from HEK293 was used for AAV vector preparation.
Authentication	The cell lines were not authenticated.
Mycoplasma contamination	The cell line was not tested for mycoplasma contamination.
Commonly misidentified lines (See ICLAC register)	None of the cell lines we used is listed in the database of commonly misidentified cell lines maintained by ICLAC.

Animals and other organisms

Policy information about studies involving animals; ARRIVE guidelines recommended for reporting animal research

Laboratory animals	Wistar rat, embryonic day 18 were used for the preparation of hippocampal prymary neurons. Adult C57BL/6J mice and Long-Evans rats were used for immunohistochemistry and electrophysiology.
Wild animals	This study did not involve wild animals.
Field-collected samples	This study did not involve samples collected from the field.
Ethics oversight	the care and use of experimental animals and approved by the Institutional Committee for the Care and Use of Experimental Animals at the University of Yamanashi (protocol # A30-21), by the Institutional Animal Care and Use Committee of Tokyo Medical and Dental University (protocol #A2019-274), and by the Institutional Animal Care and Use Committee of the Jikei University (protocol #2017-048) and conformed to the Guidelines for the Proper Conduct of Animal Experiments of the Science Council of Japan (2006).

Note that full information on the approval of the study protocol must also be provided in the manuscript.
